# Targeted mitochondrial therapy using MitoQ shows equivalent renoprotection to angiotensin converting enzyme inhibition but no combined synergy in diabetes

**DOI:** 10.1038/s41598-017-15589-x

**Published:** 2017-11-09

**Authors:** Micheal S. Ward, Nicole B. Flemming, Linda A. Gallo, Amelia K. Fotheringham, Domenica A. McCarthy, Aowen Zhuang, Peter H. Tang, Danielle J. Borg, Hannah Shaw, Benjamin Harvie, David R. Briskey, Llion A. Roberts, Manuel R. Plan, Michael P. Murphy, Mark P. Hodson, Josephine M. Forbes

**Affiliations:** 1 Glycation and Diabetes Group, Mater Research Institute-The University of Queensland, Translational Research Institute, Woolloongabba, Queensland Australia; 2 Schools of Biomedical Sciences, Woolloongabba, Queensland Australia; 3 Medicine, Schools of Biomedical Sciences, Woolloongabba, Queensland Australia; 4 Human Movement and Nutrition Sciences, St Lucia, Queensland Australia; 5 Pharmacy The University of Queensland, St Lucia, Queensland Australia; 6 Department of Paediatrics, University of Cincinnati, Cincinnati, Ohio, USA; 7 The University of Queensland Biological Resources, St Lucia, Queensland Australia; 8 Metabolomics Australia Queensland Node, Australian Institute for Bioengineering and Nanotechnology, The University of Queensland, St Lucia, Queensland Australia; 9 MRC Mitochondrial Biology Unit, University of Cambridge, Cambridge, UK; 10 Department of Medicine, The University of Melbourne, Heidelberg, Australia

## Abstract

Mitochondrial dysfunction is a pathological mediator of diabetic kidney disease (DKD). Our objective was to test the mitochondrially targeted agent, MitoQ, alone and in combination with first line therapy for DKD. Intervention therapies (i) vehicle (D); (ii) MitoQ (DMitoQ;0.6 mg/kg/day); (iii) Ramipril (DRam;3 mg/kg/day) or (iv) combination (DCoAd) were administered to male diabetic *db*/*db* mice for 12 weeks (*n* = 11–13/group). Non-diabetic (C) *db*/*m* mice were followed concurrently. No therapy altered glycaemic control or body weight. By the study end, both monotherapies improved renal function, decreasing glomerular hyperfiltration and albuminuria. All therapies prevented tubulointerstitial collagen deposition, but glomerular mesangial expansion was unaffected. Renal cortical concentrations of ATP, ADP, AMP, cAMP, creatinine phosphate and ATP:AMP ratio were increased by diabetes and mostly decreased with therapy. A higher creatine phosphate:ATP ratio in diabetic kidney cortices, suggested a decrease in ATP consumption. Diabetes elevated glucose 6-phosphate, fructose 6-phosphate and oxidised (NAD+ and NADP+) and reduced (NADH) nicotinamide dinucleotides, which therapy decreased generally. Diabetes increased mitochondrial oxygen consumption (OCR) at complex II-IV. MitoQ further increased OCR but decreased ATP, suggesting mitochondrial uncoupling as its mechanism of action. MitoQ showed renoprotection equivalent to ramipril but no synergistic benefits of combining these agents were shown.

## Introduction

There is a rising global incidence of diabetes where progressive diabetic kidney disease (DKD) seen in 25–40% of individuals, is a major factor driving mortality risk^1^. Inhibitors of the renin-angiotensin system are first line therapies administered upon clinical presentation of DKD^2^. Although recent figures suggest a stabilisation in the prevalence of DKD^3^, current therapies only slow progression of the disease and transplantation or dialysis is ultimately required.

With diabetes, it is postulated that tissues with greater metabolic demand are at risk of chronic complications^4,5^. The kidney cortex has high requirements for aerobic adenosine triphosphate (ATP) synthesis via oxidative phosphorylation^6^, due to processes such as tubular reabsorption of glucose, ions and other metabolites from the urinary filtrate by tubules^7,8^, and control of glomerular filtration^9^. As such, the kidneys contain many mitochondria^10^ and at rest are second only to the heart in oxygen consumption when considered by organ weight^6^. In diabetes, metabolism^11,12^, oxygen consumption^13–15^ and glomerular ATP concentrations^9^ are increased early in disease pathogenesis.

Mitochondrial dysfunction is seen early in the development of experimental DKD^13,16^ and has been identified as a major contributor to disease progression both in preclinical models^13,17,18^ and in humans with DKD^19–21^. There is also evidence of mitochondrial dysfunction in other chronic kidney diseases^22–24^. Further, mitochondrial dysfunction can confer susceptibility to chronic kidney disease (CKD)^24–26^. Therapies thought to improve mitochondrial function, including Coenzyme Q10 (CoQ10)^18,27,28^ and SS-31 have beneficial effects on kidney function and fibrosis in experimental models of diabetes^29^ and obesity^30^.

MitoQ is a form of coenzyme Q with a lipophilic cation that selectively facilitates its uptake into the mitochondria where it is postulated to act as an anti-oxidant. MitoQ has demonstrated consistent benefits in disease settings^31,32^ and is under investigation in CKD Stages 3–5 (NCT02364648). To date, only one study has explored the utility of MitoQ in treating DKD, where daily administration prevented albuminuria in an experimental model of monogenic diabetes of the young (MODY), the *Ins2* ^*Akita*^ mouse^17^.

Therefore, in the present study, our objective was to compare the renoprotection afforded by MitoQ as a monotherapy and in combination with a first line therapy for DKD, the angiotensin-converting enzyme (ACE) inhibitor, ramipril. The ways in which MitoQ and ramipril differed in conferring renoprotection in the diabetic kidney were also investigated.

## Results

### Body mass and metabolic parameters

**Figure 1:**
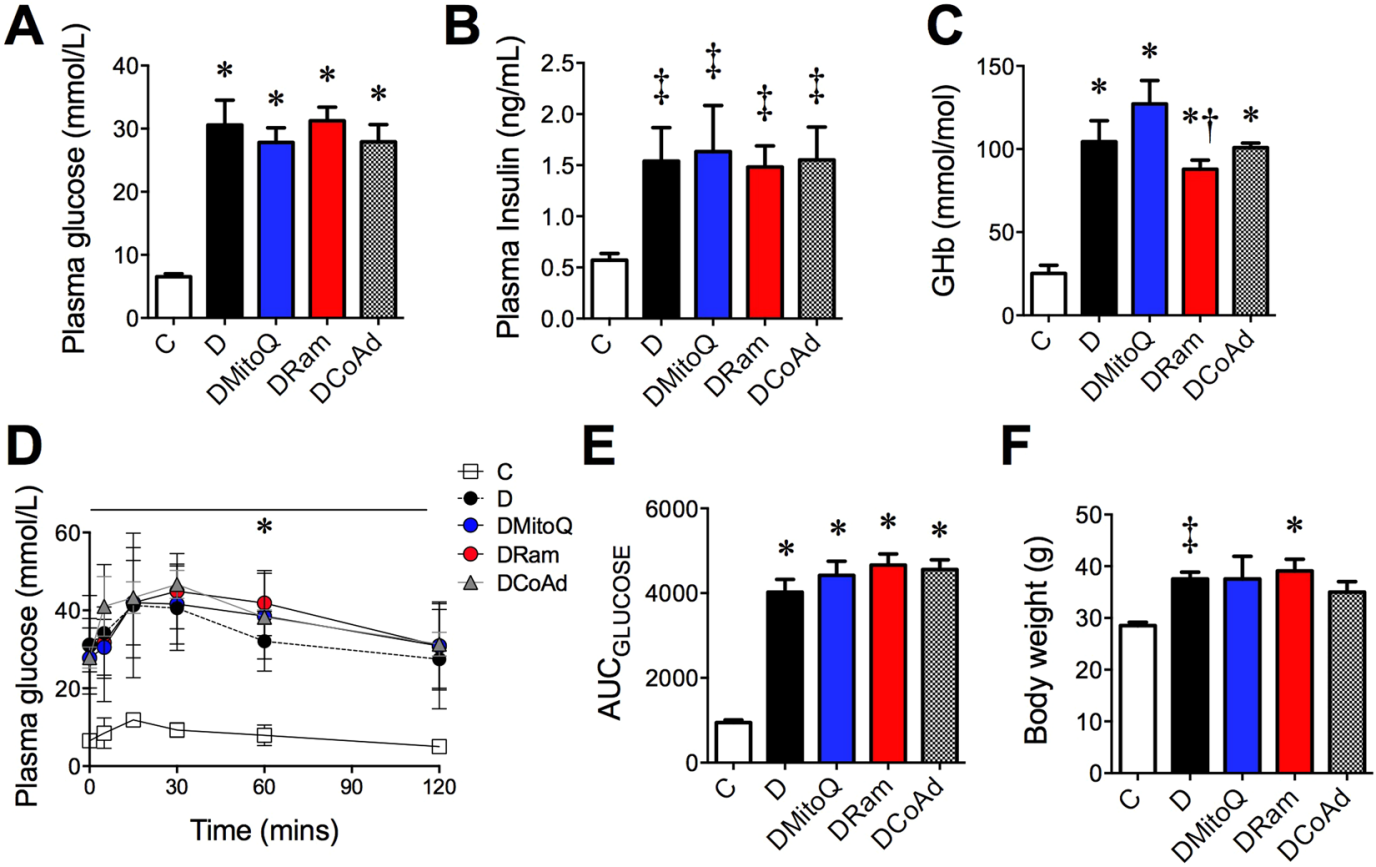
Therapeutical intervention with MitoQ, the ACE inhibitor rampril or their combination, does not affect body mass or glucose homeostasis in experimental type 2 diabetes. White bars/circles – Non-diabetic *db*/*m* mice (**C**); Black bars/circles − diabetic *db*/*db* mice (**D**); Blue bars/circles − diabetic *db*/*db* mice + 0.6 mg/kg/day MitoQ intragastrically (DMitoQ); Red bars/circles - diabetic *db*/*db* mice + 3 mg/kg/day ramipril intragastrically (DRam); Grey bars/circles - diabetic *db*/*db* mice + combination of MitoQ and ramipril (DCoAd). Therapies were adminstered for 12 weeks. n = 6–13 mice/group. (**A**) Fasted plasma glucose concentrations; (**B**) Fasted plasma insulin concentrations; (**C**) Glycated hemoglobin concentrations; (**D**) Timecourse and (**E**) AUC plasma glucose concentrations following an intragastric 2 mg/kg D-glucose bolus. (**F**) Body weight. All data are expressed as mean ± SD. *P < 0.05 vs C by 1 W ANOVA/Tukey’s Post-hoc; ^†^ *P* < 0.05 vs DMito 1 W ANOVA/Tukey’s Post-hoc; ^‡^ *P* < 0.05 vs C by Student’s unpaired t-test.

At study commencement, all diabetic mice were overweight, with elevated fasting plasma glucose and glycated hemoglobin concentrations, when compared with non-diabetic mice (Table 1). By the study end, fasting plasma glucose (Fig. 1A) insulin (Fig. 1B), and glycated hemoglobin concentrations (Fig. 1C) were all markedly increased by diabetes, but not affected by therapy. However, ramipril treated mice had lower glycated hemoglobin concentrations when compared with MitoQ monotherapy (Fig. 1C). All diabetic mice had glucose intolerance, determined by an oral glucose tolerance test (OGTT), which was not altered by therapy (Fig. 1D,E). All diabetic mice remained overweight at the study end and this was not different among groups (Fig. 1F). Mice with diabetes consumed more food and water and had greater urine output throughout the study and these were unaffected by therapy (Table 1).

**Table 1:** Baseline and study end anthropometric and biochemical parameters for diabetic (*db*/*db*) and non-diabetic (*db*/*m*) mice.

	Control	Diabetes	DMitoQ	DRam	DCoAd
**At study commencement**					
Body wt (g)	25.4 ± 1.5	39.1 ± 1.4*	37.2 ± 2.5*	36.2 ± 2.5*	40.0 ± 1.8*
FBG (mmol/L)	9.1 ± 2.6	23.8 ± 4.8*	29.3 ± 7.0*	22.2 ± 4.1*	24.5 ± 4.5*
GHb (%)	5.7 ± 1.9	7.8 ± 2.5*	7.4 ± 1.6*	6.7 ± 2.9*	7.0 ± 1.9*
**At study end**					
Kidney weight (mg/mm tibial length)	14.8 ± 1.4	20.8 ± 2.1*	19.8 ± 3.7*	20.1 ± 2.7*	22.2 ± 2.3*
Food consumption (g/24 h)	5.3 ± 1.1	9.7 ± 2.2*	8.4 ± 2.9*	9.8 ± 1.5*	9.8 ± 3.1*
Water consumption (ml/24 h)	4.1 ± 0.9	21.2 ± 6.3*	19.7 ± 9.3*	23.3 ± 6.5*	24.0 ± 7.5*
Urine output (ml/24 h)	0.4 ± 0.3	15.7 ± 7.8*	13.1 ± 7.1*	16.1 ± 7.6*	17.9 ± 9.6*

Non-diabetic *db*/*m* mice (Control); Diabetic *db*/*db* mice (Diabetes); Diabetic *db*/*db* mice + 0.6 mg/kg/day MitoQ orally (DMitoQ); Diabetic *db*/*db* mice + 3 mg/kg/day Ramipril orally (DRam); Diabetic + 0.6 mg/kg/day MitoQ orally (DMitoQ) + 3 mg/kg/day Ramipril orally (DCoAd); Grey bars/dots − diabetic *db*/*db* mice + combination of MitoQ and Ramipril (DCoAd). n = 6–13 mice/group. FBG – fasting blood glucose; GHb – glycated haemoglobin; KW – kidney weight. Data expressed as Mean ± SEM apart from Body wt and KW. *P < 0.05 vs C by 1 W ANOVA/Tukey’s Post-hoc.

### Renal function and structure

**Figure 2:**
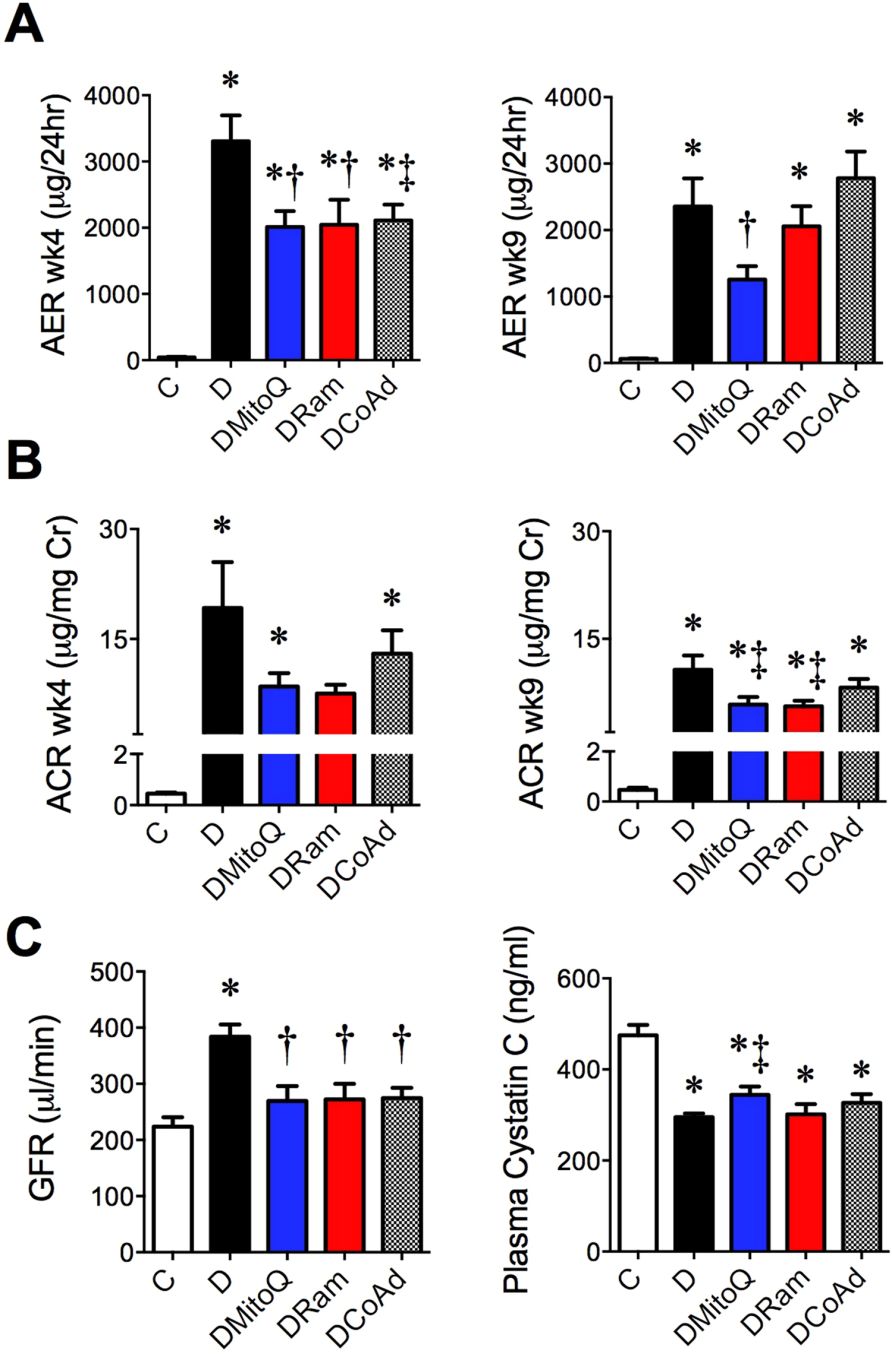
Once daily administration of MitoQ provides equivalent renoprotection to ACE inhibition in experimental diabetes, but combination therapy does not confer superior renoprotection. White bars/squares − Non-diabetic *db*/*m* mice (**C**); Black bars/dots - diabetic *db*/*db* mice (**D**); Blue bars/dots − diabetic *db*/*db* mice + 0.6 mg/kg/day MitoQ intragastrically (DMitoQ); Red bars/dots − diabetic *db*/*db* mice + 3 mg/kg/day ramipril intragastrically (DRam); Grey bars/dots - diabetic *db*/*db* mice + combination of MitoQ and ramipril (DCoAd). n = 6–13 mice/group. (**A**) Twenty four hour urinary albumin excretion rate (AER) early (4 weeks; left) and later (9 weeks, right) after treatment commenced. (**B**) Urinary albumin:creatinine ratio (ACR) at four (left) and nine (centre) weeks after treatment commenced. (**C**) Glomerular filtration rate (GFR) determined by transcutaneous decay of FITC-sinistrin dosed by body mass (left) and plasma cystatin C concentration at the study end (right). All data are expressed as mean ± SD or median ± interquartile range (cystatin C and ACR) when non-parametric. *P < 0.05 vs C by 1 W ANOVA/Tukey’s Post-hoc or Kruskall Wallis/Dunn’s Post hoc; ^†^ *P* < 0.05 vs D by 1 W ANOVA/Tukey’s Post-hoc or Kruskall Wallis/Dunn’s Post hoc; ^‡^ *P* < 0.05 vs D by Student’s unpaired t-test.

Diabetic mice had significant albuminuria, shown by elevations in 24 h urinary albumin excretion rate (AER; Fig. 2A) and urinary albumin creatinine ratio (ACR; Fig. 2B) both early (week 4) and later (week 9) during the development of DKD. Early in disease, MitoQ, ramipril and their combination attenuated albuminuria assessed by AER, but not ACR in diabetic mice (Fig. 2A,B, left). Later in disease development, MitoQ attenuated the diabetes-induced increases in AER, but this was not seen with ramipril or combination therapy (Fig. 2A, right). However, both MitoQ or ramipril monotherapy modestly decreased the diabetes-induced increases in ACR later in DKD (Fig. 2B, right).

By the study end, diabetic mice which did not receive therapy had a ~two-fold increase in GFR compared with non-diabetic mice (Fig. 2C, left). This was supported by a decrease in plasma cystatin C with diabetes (Fig. 2C, right). The diabetes-induced increase in GFR was ameliorated by all therapies (Fig. 2C, left). However, plasma cystatin C concentrations in diabetic mice were only increased by MitoQ monotherapy (Fig. 2C, right).

**Figure 3:**
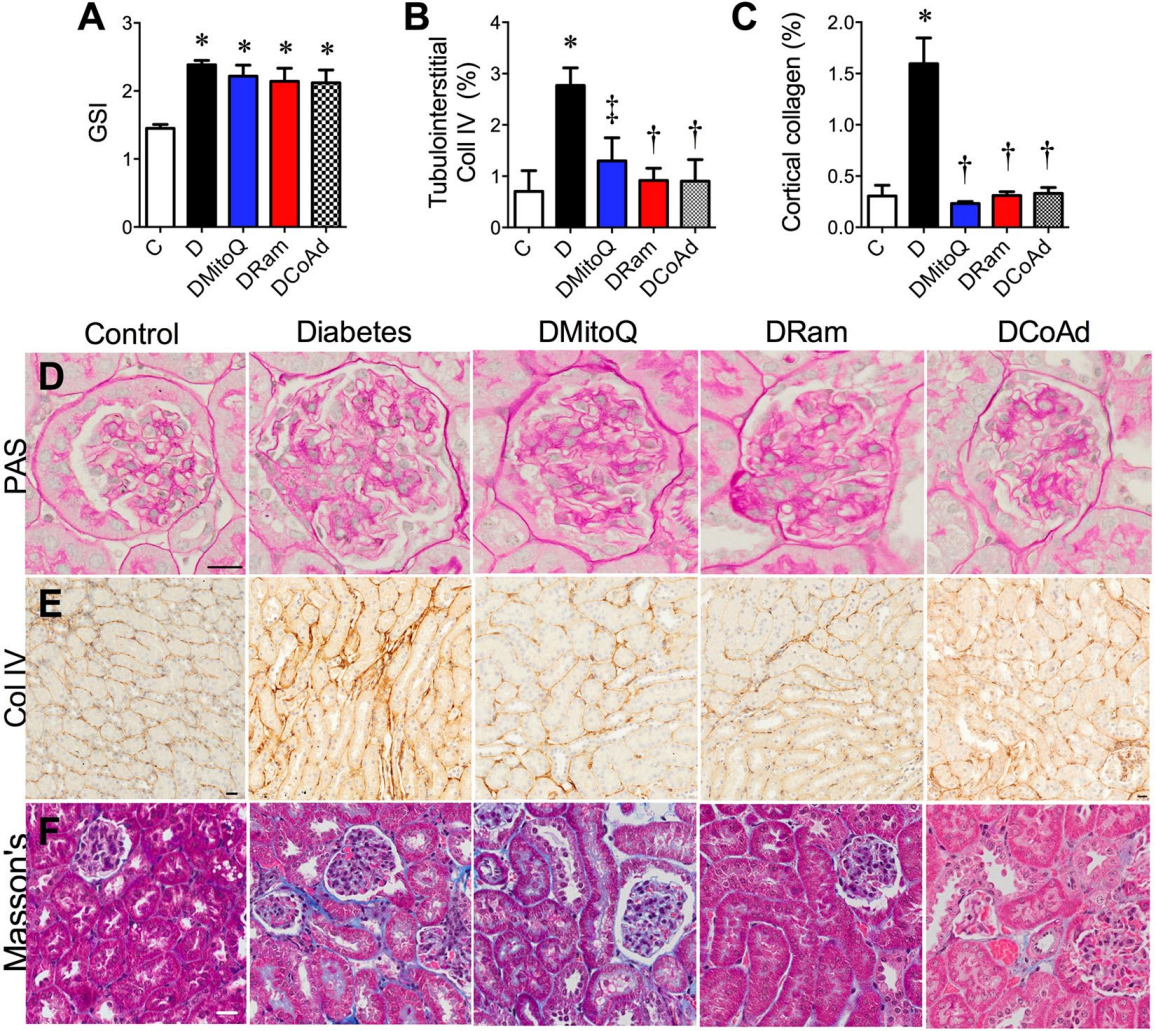
MitoQ administration improves tubulointerstitial fibrosis in diabetic (*db*/*db*) mice but combination therapy is not superior. White bars – Non-diabetic *db*/*m* mice (**C**); Black bars − diabetic *db*/*db* mice (**D**); Blue bars − diabetic *db*/*db* mice + 0.6 mg/kg/day MitoQ intragastrically (DMitoQ); Red bars − diabetic *db*/*db* mice + 3 mg/kg/day ramipril intragastrically (DRam); Grey bars/dots - diabetic *db*/*db* mice + combination of MitoQ and ramipril (DCoAd). n = 6 mice/group). At the study end, glomerular injury was quantified by (**A)** Periodic Acid Schiff (PAS) staining and assessment of glomerulosclerosis (GSI). Tubulointerstitial injury was assessed by (**B**) Immunohistochemistry for collagen IV (Coll IV). (**C**) Masson’s Trichrome collagen staining. Representative photomicrographs of renal cortical structural markers; (**D**) PAS (x400); (**E**) Coll IV (x200) (**F**) Masson’s Trichrome. Scale bar = 20 μm. All data are expressed as mean ± SEM. *P < 0.05 vs C by 1 W ANOVA/Tukey’s Post-hoc; ^†^ *P* < 0.05 vs D by 1 W ANOVA/Tukey’s Post-hoc; ^‡^ *P* < 0.05 vs D by Student’s t test.

All diabetic mice had renal hypertrophy (Table 1) and mesangial expansion (Fig. 3A; *Pictured 3D*) by the end of the study, irrespective of the therapeutic intervention. Tubulointerstitial collagen IV deposition was ~three-fold greater in diabetic versus non-diabetic mice, and this was ameliorated by all therapies (Fig. 3B; *Pictured 3E*). Mice with diabetes also had increased cortical staining of tubulointerstitial collagen using Masson’s Trichrome (Fig. 3C, *Pictured 3 F*), which was also alleviated by each therapy.

### Renal cortical energy storage and substrate metabolites

**Figure 4:**
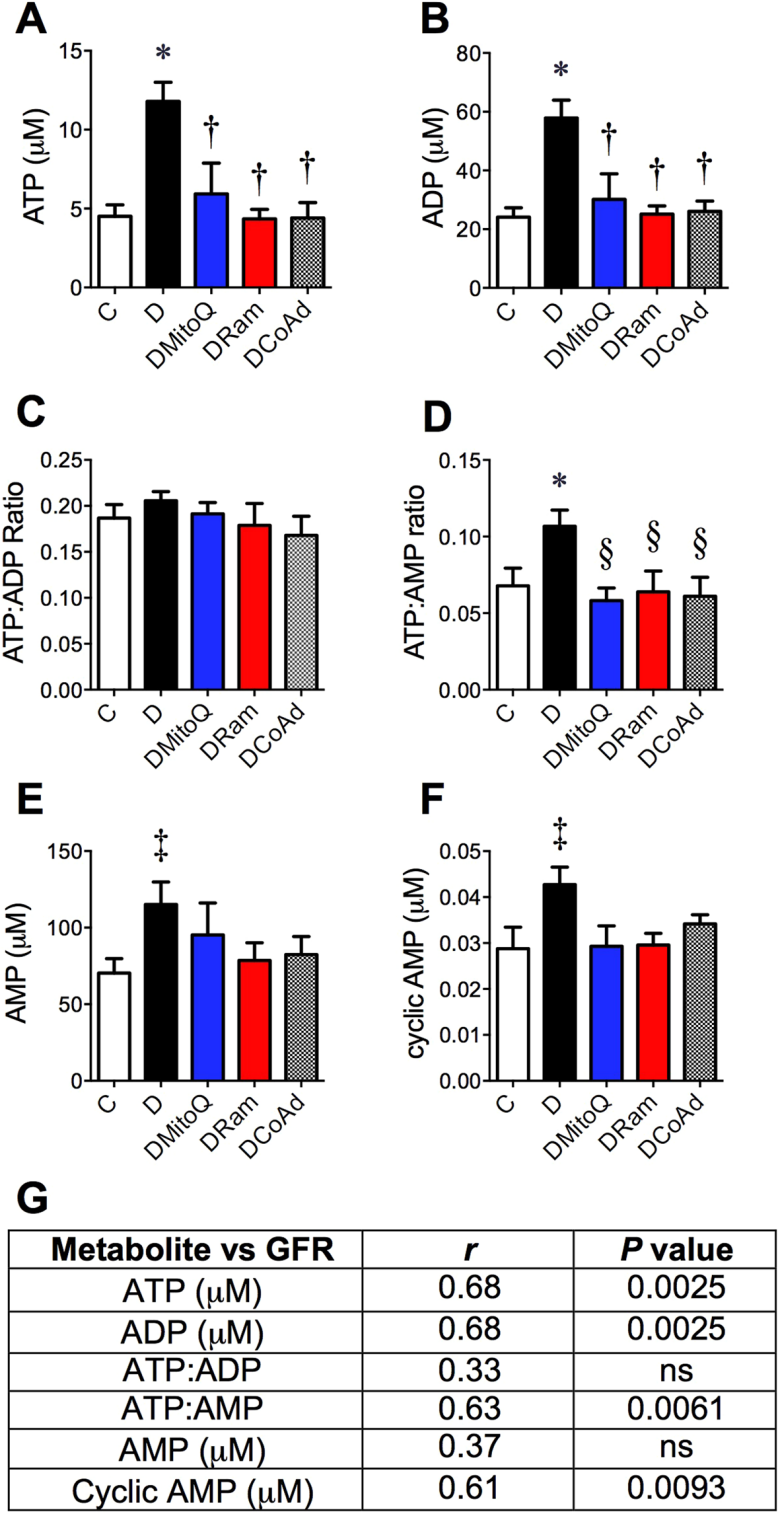
Renal cortical concentrations of adenine based nucleotides relate to glomerular filtration. White squares − Non-diabetic *db*/*m* mice (**C**); Black dots - diabetic *db*/*db* mice (**D**); Blue dots − diabetic *db*/*db* mice + 0.6 mg/kg/day MitoQ intragastrically (DMitoQ); Red dots − diabetic *db*/*db* mice + 3 mg/kg/day ramipril intragastrically (DRam); Grey bars/dots - diabetic *db*/*db* mice + combination of MitoQ and ramipril (DCoAd). n = 6 mice/group. Renal cortical concentrations of (**A)** adenosine triphosphate (ATP); (**B**) adenine diphosphate (ADP); (**C**) ATP:ADP ratio; (**D**) ATP: adenosine monophosphate ratio (ATP:AMP); (**E**) AMP; (**F**) cyclic AMP; (**G**) Table of Pearson’s correlation coefficients for associations between renal function (FITC sinistrin based GFR) and renal concentrations of adenine molecules. ns – not significant. *P < 0.05 vs C 1 W ANOVA/Tukey’s Post-hoc; ^†^ *P* < 0.05 vs D by 1 W ANOVA/Tukey’s Post-hoc; ^‡^ *P* < 0.05 vs C by Student’s unpaired t test; §*P* < 0.05 vs D by Student’s unpaired t test.

Energy production including concentrations of adenosine based molecules can mediate glomerular filtration^33^. Diabetes increased the renal cortical concentrations of the adenosine based energy storage molecules adenosine tri- (ATP; Fig. 4A) and diphosphate (ADP; Fig. 4B) but did not alter the ATP:ADP ratio (ATP:ADP; Fig. 4C). Increases in renal cortical ATP:adenosine monophosphate ratio (ATP:AMP; Fig. 4D), AMP (Fig. 4E) and cyclic AMP (ADP; Fig. 4F) concentrations were also seen with diabetes. Therapeutic intervention decreased both ATP (Fig. 4A) and ADP (Fig. 4B) concentrations as well as the ATP:AMP ratio (Fig. 4D) in diabetic kidney cortices, but did not affect other adenosine based molecules. The renal cortical concentrations of ATP, ADP, cAMP and the ATP:AMP ratio were each significantly related to the glomerular filtration rate (GFR; Fig. 4G) but not albuminuria (AER; coefficients not shown). Overall there were no significant differences among groups in the ratio of creatine phosphate:ATP (C, 1.2 ± 0.4 vs D, 2.5 ± 0.7; *P* = 0.0 and DMitoQ, 2.3 ± 2.3, DRam, 2.6 ± 1.6, DCoAd, 2.6 ± 1.6).

**Figure 5:**
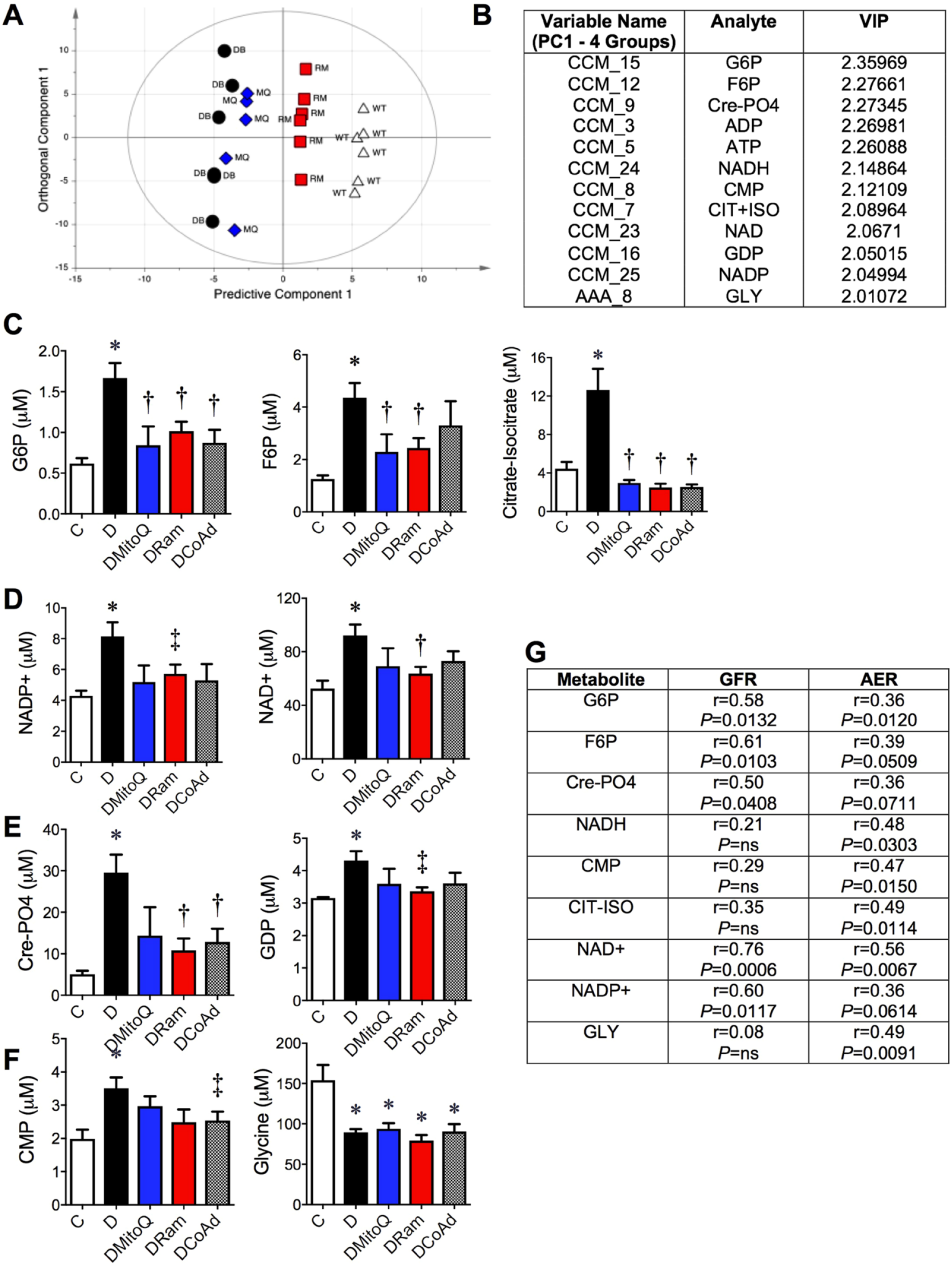
The renal metabolomic profile of central carbon metabolites and amino acids associates with renal dysfunction in diabetes. White bars/ squares − Non-diabetic *db*/*m* mice (**C**); Black bars/dots diabetic *db*/*db* mice (**D**); Blue bars/triangles − diabetic *db*/*db* mice + 0.6 mg/kg/day MitoQ intragastrically (DMitoQ); Red bars/squares diabetic *db*/*db* mice + 3 mg/kg/day ramipril intragastrically (DRam); Grey bars/dots - diabetic *db*/*db* mice + combination of MitoQ and ramipril (DCoAd). n = 6 mice/group. (**A**) Bioinformatic scores plot of the OPLS-DA statistical bioinformatic model of all metabolomics data, which demonstrates clear separation among mouse groups along the predictive component axis (*x* axis), with intra-group variability along the orthogonal (*y* axis). (**B**) Table of the most influential metabolites which define the differences between diabetic (**D**) and all other mouse groups. **b-f** Renal cortical concentrations of (**C**) glycolytic/gluconeogenic intermediates glucose 6-phosphate (G6P, left), fructose 6-phosphate (F6P) and the TCA cycle intermediates citrate-isocitrate (right); (**D**) oxidised nicotinamide adenine dinucleotides NADP+ (left) and (NAD+, right); (**E**) the energy storage molecules creatinine phosphate (Cre-PO_4,_ left), guanosine diphosphate (GDP, right) and (**F**) cytidine monophsophate (CMP, left); and the amino acid glycine (right). (**G**) Table of Pearson’s correlation coefficients for associations between renal function (GFR and 24 h urinary AER) and the renal cortical metabolites. CCM- central carbon metabolite; AAA- amino acid analyte. *P < 0.05 vs C by 1 W ANOVA/Tukey’s Post-hoc; ^†^ *P* < 0.05 vs D by 1 W ANOVA/Tukey’s Post-hoc; ^‡^ *P* < 0.05 vs D by Student’s unpaired t test.

Central carbon metabolism (CCM) uses a complex series of enzymatic steps to convert nutrients into metabolic precursors for energy production within cells. Analyses of renal cortical central carbon metabolites and amino acid content were performed (See Supplementary Tables S1–S2 for all metabolites). By applying multivariate analysis (Fig. 5A, OPLS-DA scores plot), the metabolites which best predicted the differences between the untreated diabetic group and all other mouse groups were determined. The highest ranked metabolites predicting the differences between the *db*/*db* diabetic (D) mouse group and all other mouse groups are shown (Fig. 5B). The variable importance on prediction (VIP) parameter shown in the table (Fig. 5B) ranks the metabolites in order of influence on the multivariate model.

Examination of the specific metabolites ranked by this model showed that the renal cortical concentrations of two glycolytic/gluconeogenic metabolites, glucose 6-phosphate (G6P; Fig. 5C, left) and fructose 6-phosphate (F6P; Fig. 5C, centre), were increased by diabetes and ameliorated with either monotherapy. There was a positive association between renal cortical G6P and F6P concentrations and renal function, defined both by GFR and 24 h urinary AER (Fig. 5G). The tricarboxylic acid (TCA) intermediate citrate-isocitrate (Fig. 5C, right) as well as the electron acceptors, oxidised nicotinamide adenine dinucleotide (NAD+ and NADP+), were also increased in diabetic kidney cortices (Fig. 5D). Ramipril decreased the renal oxidised isoforms, NAD+ and NADP+, when compared with diabetic mice (Fig. 5D), but MitoQ and combination therapy only trended towards decreasing renal NADP+ (*P* = 0.082) concentrations. Both oxidised NAD+ and reduced NADH were significantly associated with GFR and AER (Fig. 5G). Renal cortical NADP+ concentrations were related only to GFR and not AER (Fig. 5G). Diabetes increased renal concentrations of cytidine monophosphate (CMP; Fig. 5F, left) and decreased the amino acid glycine (GLY; Fig. 5F, right) and these were related to AER (Fig. 5G), but were unaffected by therapy (Supplementary Data, Tables S1 and S2).

### Differences between MitoQ and Ramipril

**Figure 6:**
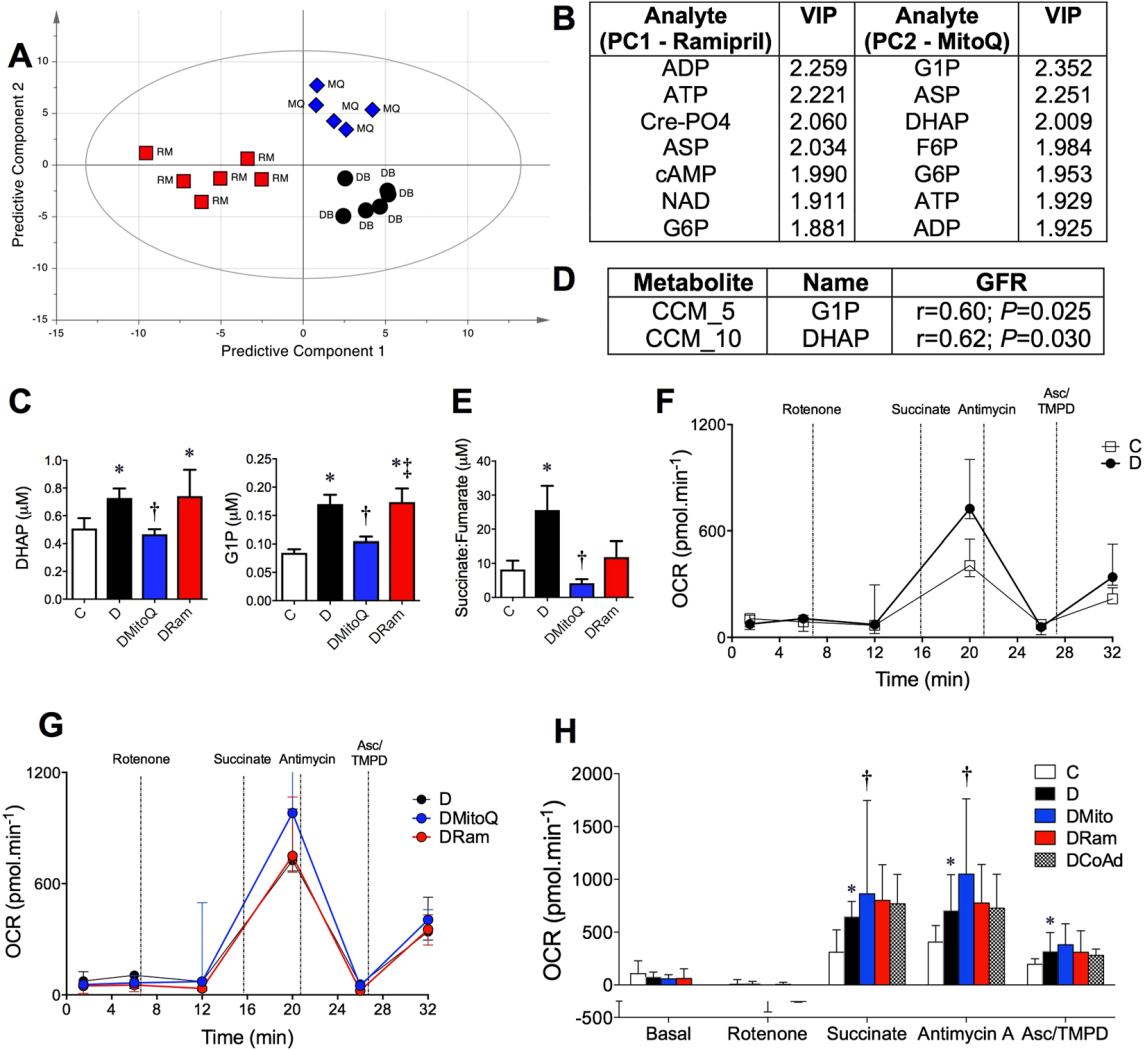
Differences between MitoQ and rampril treated diabetic kidney cortices. White bars – Non-diabetic *db*/*m* mice (**C**); Black bars/dots diabetic *db*/*db* mice (**D**); Blue bars/triangles diabetic *db*/*db* mice + 0.6 mg/kg/day MitoQ intragastrically (DMitoQ); Red bars/squares diabetic *db*/*db* mice +3 mg/kg/day ramipril intragastrically (DRam); Grey bars/dots - diabetic *db*/*db* mice + combination of MitoQ and ramipril (DCoAd). n = 6 mice/group. (**A**) Scores plot of the bioinformatic (OPLS-DA) model including all renal metabolites, showing clear separation among diabetic mouse groups where the predictive component 1 axis is for untreated diabetic (**D**) mice vs ramipril treated mice are shown (*x* axis, PC1), plotted against the predictive component 2 axis for untreated diabetic mice vs MitoQ treated mice (*y* axis, PC2). (**B**) Table of the most influential metabolomic variables which define the differences between treated and untreated diabetic mouse groups. The variable importance on prediction (VIP) parameter ranks the metabolites in order of significance. Renal cortical concentrations of the metabolites which differ between MitoQ and ramipril treated mouse groups, (**C**) dihydroxyacetone phosphate (DHAP) and glucose 1-phosphate (G1P), (**D**) Pearson’s correlations between renal function defined as GFR and G1P/DHAP in all mice. (**E**) succinate:fumarate ratio, **F-h** SeaHorse XF24 Flux Analyses of oxygen consumption rate (OCR) during a maximal electron flow test in isolated uncoupled renal cortical mitochondria in the presence of FCCP and the complex I substrates, pyruvate and malate. n = 3 in the *db*/*m* and n = 5 mice/group in the *db*/*db* with and without treatment. Line traces of OCR are shown for (**F**) control vs diabetic untreated mice and (**G**) diabetic MitoQ vs ramipril treated mouse groups - the curve for the diabetic untreated group sits below the ramipril group. (**H**) Mitochondrial OCR responses at baseline, following addition of rotenone (CI inhibitor), succinate (CII substrate), antimycin A (CIII) inhibitor and ascorbic acid + TMPD − N,N,N′,N′-tetramethyl-p-phenylenediamine (Asc/TMPD; CIV substrates) of the mitochondrial respiratory chain. For electron flow, all data are expressed as median ± interquartile range. *P < 0.05 vs C; ^†^ *P* < 0.05 vs D; ^‡^ *P* < 0.05 vs DMitoQ all by 1 W ANOVA/Tukey’s Post-hoc.

A multivariate statistical model including treated and untreated diabetic mice was constructed to examine differences between MitoQ and rampril. A representative OPLS-DA scores plot of this model is shown (Fig. 6A), highlighting the relative biochemical similarities and differences among the groups. The major metabolites predicting the differences between MitoQ and ramipril therapy in *db*/*db* diabetic mice are shown (Fig. 6B). Diabetes increased the concentrations of dihydroxyacetone phosphate (DHAP; Fig. 6C, left), a precursor for glycerol 3-phosphate and synthesis of other fatty acids and glucose 1-phosphate (G1P; Fig. 6C, right), a molecule produced during glycogen breakdown by glycogenolysis. MitoQ, but not ramipril, therapy attenuated the diabetes-induced increases in renal cortical DHAP and G1P (Fig. 6C). The changes in DHAP and G1P were associated with GFR (Fig. 6D) but not urinary AER (coefficients not shown). The ratio of succinate to fumarate, a surrogate measure of complex II function was increased by diabetes and this was attenuated by MitoQ but not ramapril monotherapy (Fig. 6E).

Evidence from *in vitro* studies suggests that MitoQ may act as an electron carrier by accepting electrons from complexes I and II and donating these to complex III, to improve mitochondrial and cellular function^34–36^. Hence, mitochondrial function was examined using a maximal electron flow test measuring oxygen consumption rate (OCR) in uncoupled mitochondria during inhibition of various respiratory chain complexes. Here, the flow of electrons from complex I to complex IV is monitored in the presence of FCCP, an uncoupler that carries protons away through the inner membrane without formation of a proton gradient or ATP. Therefore, defects in a specific complex can monitor changes in oxygen consumption when a complex-specific substrate is provided. A representative trace during the electron flow assay shows that diabetes increased oxygen consumption in renal cortical mitochondria when compared with mitochondria from control kidney cortices (Fig. 6F). There were increases in oxygen consumption during the electron flow test in diabetic kidney mitochondria following inhibition of complex I and addition of the complex II substrate, succinate (Fig. 6F and H). Increased oxygen consumption by mitochondria from the diabetic kidney was also seen with inhibition of complex III (Antimycin A) and following donation of electrons directly to complex IV (Ascorbate/TMPD). There were no differences in oxygen consumption in uncoupled mitochondria from diabetic versus control kidney cortices at baseline or following complex I inhibition (Rotenone, Fig. 6G).

MitoQ monotherapy specifically increased the OCR (Fig. 6F) above that seen in mitochondria taken from vehicle treated diabetic mice following inhibition of complex I and addition of the complex II substrate, succinate (Fig. 6G). Increases in OCR with MitoQ monotherapy in mitochondria from diabetic mice were also seen with inhibition of complex III (Fig. 6G), when compared with mitochondria from untreated diabetic mice (Fig. 6E). In the presence of decreased ATP content and unchanged creatine phosphate to ATP ratio, this suggested that MitoQ was acting via mitochondrial uncoupling. MitoQ therapy did not influence OCR during the electron flow test at baseline, following complex I inhibition nor following donation of electrons to complex IV (Fig. 6F,G). Ramipril did not alter oxygen consumption during mitochondrial electron flow testing in diabetic mice (Fig. 6F,G).

### Oxidised and reduced coenzyme Q9 and Q10 in the kidney

Since MitoQ is a coenzyme Q derivative with a lipophilic cation to facilitate trafficking to mitochondria, we assessed whether its mechanism of action included effects on mitochondrial concentrations of Coenzyme Q10 (CoQ10). CoQ10 plays a central role in trafficking electrons between complexes I, II and III during mitochondrial electron transport. Renal concentrations of oxidised (ubiquinone) and reduced (ubiquinol) CoQ9 and CoQ10 were analysed by HPLC both in cortical homogenates and isolated mitochondria. At study end, total CoQ9 content in cortical tissue was increased in all *db*/*db* diabetic mice irrespective of therapeutic intervention (Supplementary Data, Table S3). Total CoQ9 to Q10 ratio was also significantly increased by diabetes and not altered by therapy (Supplementary Data, Table S3). Cortical and mitochondrial ubiquinol content did not change with diabetes nor therapeutic intervention (Supplementary Data, Table S3).

## Discussion

In the present study, we demonstrate that once daily oral administration of MitoQ for 12 weeks improved renal function (albuminuria, hyperfiltration) and attenuated tubulointerstitial pathology (collagen) with equivalent efficacy to oral ACE inhibition using ramipril. However there were no synergistic benefits seen with their combined administration. The diabetic kidney had elevations in the cortical concentrations of the metabolites of oxidative phosphorylation and the purine nucleotides ATP, ADP, cyclic AMP and the ratio of ATP:AMP, as well as the phosphate storage molecule and phosphate/ATP buffering molecule, Cre-PO_4_. All therapeutic interventions attenuated the increases in ATP, ADP and the ATP:AMP ratio. Across all groups, GFR positively correlated with cortical concentrations of the purine nucleotides ATP, ADP, cyclic AMP, the ratio of ATP:AMP and Cre-PO_4_. Interestingly, there was no discernible relationship between albuminuria by AER and the concentrations of these phosphate fuel storage molecules in kidney cortices. Oxidised (NAD+ and NADP+) and reduced (NADH) nicotinamide dinucleotide concentrations were elevated by diabetes in renal cortices and related to both GFR and AER, irrespective of group. Mechanistically, MitoQ differed from ramipril in that it appeared to act as an uncoupler increasing mitochondrial oxygen consumption and limiting ATP production, creating a futile cycle where excess energetic flux through the respiratory chain was dissipated as heat. MitoQ also attenuated diabetes-induced increases in the glycerol/fatty acid synthesis precursor, DHAP and the breakdown product of glycogenolysis, glucose-1-phosphate. The diabetes-induced mesangial matrix expansion was not attenuated with either mono- or combination therapy.

Previous studies have identified mitochondrial dysfunction^13,17,19,21,27,37^, including increases in mitochondrial OCR as early pathological events in the kidneys of diabetic rodents^11,13^. In agreement, we have now shown that mitochondria isolated from kidney cortices of diabetic mice have increased OCR and electron flow specifically at complexes II through to IV of the electron respiratory chain, most likely due to impairment of complex I activity, which has been previously shown in the diabetic kidney^13,38^. In the present study, there were concomitant increases in ATP, AMP, and cyclic AMP content as well as elevated ATP:AMP ratios in diabetic kidney cortices^9,39^. Increases in production and/or turnover of renal ATP in our model, agree with a previous study in glomeruli isolated from rodent diabetic kidneys^9^. However, the higher ratio of creatine phosphate to ATP in our diabetic kidney cortices, indicate that may be the result of a decrease in ATP consumption rather than just over-production, suggesting a breakdown in energy sensing feedback loops including via ADP. Indeed, on one hand ADP content remained higher in diabetic kidney cortices, which would signal for greater ATP production, increased GFR and oxygen delivery. Conversely, however, increases in cellular ATP content could be simultaneously signalling for decreased renal ATP production and for decreased GFR in the diabetic kidney stimulating opposing signalling pathways. The accumulation of ATP, likely as the result of lower consumption of ATP rather than over production by the diabetic kidney, warrants further investigation.

Since mitochondria studied *ex vivo* from mice treated with MitoQ had greater oxygen consumption in the face of decreases in ATP, one could suggest that *in vivo* during the course of treatment, MitoQ is acting an uncoupler of the respiratory chain and ATP production which may be realigning ATP and ADP feedback pathways in the kidney, thereby restoring the GFR. Indeed, MitoQ acting *in vivo* as an uncoupler has been previously described in endothelial cells *in vitro* ^40^. However, these postulates do not explain the elevation in OCR seen with MitoQ. A more likely explanation is that MitoQ separated the rate of electron transport in the respiratory chain and oxygen consumption from ATP production, which is supported by the increase in OCR in the face of decreased ATP in the present study. Termed as energy dissipating pathways, these processes can increase heat and limit ROS generation. This could also explain why mitochondria from diabetic mice treated with MitoQ showed increased oxygen consumption above that seen in mitochondria from diabetic mice. In addition, these actions of MitoQ in the presence of ramipril, where metabolism was already limited, may explain the lack of synergy between these two agents in improving renal functional markers in this study. However, this requires examination in future studies.

Activation of pathways by diabetes which require increases in the production and accumulation of renal ATP, such as kidney reabsorption of glucose (via sodium dependent glucose transporter, SGLT2), or increases in glycolysis and flux into glucose oxidation could also explain our observed increases in OCR, fuel storage, and cofactor molecules as well as renal hyperfiltration^41^. Previous *in vivo* studies have shown that increased ATP-dependent kidney metabolism/transport, in the context of increased mitochondrial oxygen consumption, is a pathway to kidney damage^42^. In support of this, renoprotection^43^ is seen following blockade of diabetes-induced increases in renal reabsorption of glucose and sodium using SGLT2 inhibitors, which also decrease renal oxygen consumption^44,45^ and GFR^41,46^. Lowering of ATP accumulation in the cortical tubules, could also decrease tubuloglomerular feedback and hence GFR, which is known to be altered by adenine nucleotides. The feedback of high levels of both ATP and ADP in the diabetic kidney may be disrupting adenine modulation of tubuloglomerular feedback. Interestingly, most of the benefits with therapy in the present study were seen in the tubulointersitium, supporting the efficacy of both MitoQ and ramipril in this compartment, by contrast to the lack of effects on glomerular fibrosis.

In the current study, MitoQ further elevated mitochondrial OCR at complex II and III during the electron flow assay, yet attenuated glomerular hyperfiltration. Although GFR is thought to be tightly coupled to tubular oxygen consumption and transport, these data suggest that decreases in renal filtration occurred independently of decreases in cortical oxygen consumption^44^. Another study has also shown a disconnect between oxygen consumption and hyperfiltration in a model of renal damage (Laustsen). Further, as in the present study, increases in mitochondrial OCR at complex II and III in conjunction with end-organ protection has been shown with MitoQ in previous studies^36,47^. The specificity of MitoQ effects on OCR at complex II and III is puzzling. MitoQ may have altered the activity of complex II (succinate dehydrogenase) in our model, since diabetes elevated renal succinate:fumarate ratios. Increases in this ratio, which were ameliorated by MitoQ, suggest a dysfunctional complex II in diabetes. Alternatively, it is also feasible that MitoQ limited the flow of substrates from the Kreb’s cycle into OXPHOS since succinate dehydrogenase (complex II) also participates in these reactions. Indeed, this is supported by the decreases in ATP and other adenine nucleotides seen with MitoQ.

We observed increases in the glycolytic intermediates G6P, F6P, DHAP and G1P in diabetic kidney cortices, which is consistent with a previous study in a model of early kidney disease^48^. This infers a shift towards glucose oxidation including glycolytic pathways in diabetes in order to facilitate cortical demand for ATP production, despite maximal energy production via aerobic fuel production (oxidative phosphorylation) from lactate, FFAs and glutamine already occurring. This increase in glycolysis in the diabetic kidney may be stimulated by the increased accumulation of AMP. This is interesting, given that proximal tubules which constitute a vast proportion of the renal cortex, prefer nutrients other than glucose for ATP generation and have a paucity of rate limiting glycolytic enzymes^49–53^. However, the build-up of G6P and F6P in the diabetic kidney cortex could also reflect enhanced gluconeogenesis^49,52,53^ as well as the activation of glycolysis to meet ATP requirements. Whilst this is unresolved in the present study, all therapies decreased G6P and F6P concentrations in the renal cortex, warranting follow up in future studies.

It is also not clear in the present study why the therapies showed disparate effects on AER and ACR both early and later in disease, despite consistent benefits on glomerular filtration. One postulate is that later in this rodent model, insulin insufficiency as a result of secretory defects are greater which could have altered lean muscle mass^54^ and therefore urinary creatinine excretion. In addition, it is common to use repeated early morning collections to estimate ACR and to reach efficacy that is comparable to a 24 hour AER^55^. Further, albuminuria does fluctuate in response to decreases in GFR and can spontaneously regress in some diabetic individuals^56^. Indeed, this is why GFR often provides a more accurate assessment of renal function. Interestingly, GFR rather than albuminuria was more strongly correlated with renal cortical changes in ATP and other adenine based molecules in the present study. However, albuminuria more strongly related to decreases in other metabolic intermediates assessed in this study.

Taken together, these findings suggest that MitoQ confers equivalent renoprotection to a first line therapy for DKD, the angiotensin converting enzyme inhibitor, ramipril but their combination does not confer synergistic benefits. Derangements in metabolism were prominent in the diabetic kidney with excess production and/or less consumption of energetic storage molecules such as ATP, cyclic AMP and Cre-PO_4_ all strongly correlated to glomerular filtration and tubulointerstitial damage. Distinct from ramipril, MitoQ increased mitochondrial uncoupling and electron flow at complex II and III during mitochondrial respiration. The action of MitoQ as an uncoupler may also explain its lack of synergy with ramipril when they were coadministered. These findings should provide the basis for future research to better understand the relationship among delivery of metabolic substrates, mitochondrial function and renoprotection in diabetes and a rationale to further examine mitochondrial targets as treatments for DKD.

## Methods

### Experimental Model

All procedures were approved by The University of Queensland Animal Ethics Committee in accordance with guidelines from the National Health and Medical Research Council of Australia. Male BKS.Cg-*Dock7* ^*m*^ +/+ *Lepr* ^*db*^/J (*db*/*db*) mice and heterozygote littermate controls (*db*/*m*) were purchased from Jackson laboratories (stock number 000642; Bar Harbor, Maine, USA). Mice were housed in an environmentally controlled room (constant temperature 22 °C), with a 12 h light:12 h dark cycle and access to standard chow and filtered tap water *ad libitum*. At 8 weeks of age, groups (*n* = 12–13/group) of *db*/*db* mice were randomised to daily intragastric gavage of (i) vehicle (D), (ii) mitoquinone (DMitoQ; 0.6 mg/kg, mitoquinone^57^; MS010 a kind gift of Antipodean Pharmaceuticals), (iii) the angiotensin-1 converting enzyme inhibitor (ACE) inhibitor, ramipril (DRam; 3 mg/kg/day; kind gift of Sanofi-Aventis Pharmaceuticals) or their combination (DCoAd, dosages as per monotherapy) for 12 weeks. A group (*n* = 11) of non-diabetic *db*/*m* littermate control mice also received intragastric gavage of vehicle (C) for 12 weeks. Weekly body weight and fasting blood glucose (Sensocard glucometer) were monitored throughout the study.

### Biochemical Parameters

Early (four weeks) and later (nine weeks) during treatment intervention, mice were acclimatised and metabolically caged for 24 h to determine food and water intake and collect urine. Urinary albumin and creatinine assays were performed on timed urine collections^37^. Plasma glucose, insulin and glycated hemoglobin were assessed at the beginning and end of the treatment as previously described^58^. An oral glucose tolerance test in response to a 2 mg/kg D-glucose bolus was completed just before the study end at week 11 of therapy.

### Glomerular filtration rate

Just before the study end, GFR was estimated in conscious mice using the transcutaneous decay of retro-orbitally injected FITC-sinistrin (10 mg/100 g body weight dissolved in 0.9% NaCl), as previously described^58^. Plasma cystatin C was also measured by ELISA at the study end (BioVendor, Brno, Czech Republic) according to the manufacturer’s specifications^37^.

### Histology

Paraffin embedded kidney sections were used for the blinded histological assessment of renal injury. The degree of mesangial matrix expansion was determined by calculation of glomerulosclerotic index (GSI) in twenty glomeruli (x400), using 2 µm kidney sections stained with Periodic Acid Schiff (PAS) as previously described^37^. Immunohistochemistry in 4 µm kidney sections with α-collagen IV (1:100 dilution; Abcam, Cambridge, USA) was completed using the ABC Elite (Vector Labs, Burlingame, CA)^58^. Renal cortical collagen was determined using Masson’s Trichrome staining^58^. Percent of positive DAB or Masson’s staining in at least ten cortical fields (×100) per mouse were quantified using an Olympus Virtual Slide microscope and NIS Elements (Nikon) or Image J (Fiji Distribution Package) software packages.

### Metabolomics

Central carbon metabolites and amino acids were analysed by HPLC-MS/MS and HPLC-FLD respectively at the study end using triplicate samples from each mouse. Reference standards and tributylamine (puriss plus grade) were purchased from Sigma Aldrich (Sigma Aldrich, NSW, Australia). LiChroSolv acetonitrile and AR Grade acetic acid were purchased from Merck (Darmstadt, Germany) and Labscan (Gliwice, Poland), respectively. Deionised water was generated via an Elga Purelab Classic water purification unit (Veolia, France). A total of 22 amino acids (and 3 internal standards) were quantified following a previously optimized method^59^. These included Asp, Glu, Cys, Asn, Ser, Gln, His, Gly, Thr, Arg, Ala, GABA (4-aminobutyric acid), Tyr, ABU (2-aminobutyric acid), Val, Met, Nva, Trp, Phe, Ile, Orn, Leu, Lys, Sar, Pro. Standard Curve from 2–500 μM (double for Pro)

Intermediates of central carbon metabolism (CCM) were analysed following the method described in Medina-Torres *et al*.^60^ with the following modifications – sample extracts were analysed at two concentrations to increase the likelihood of detection for low abundance metabolites as well as to dilute highly abundant metabolites (e.g. AMP, lactate, malate, succinate) into range. Thus, 100 μl of sample extract were dried down in a vacuum centrifuge (Eppendorf Concentrator Plus, Eppendorf, Australia) for ~60 min with no heating using the V-AQ program. The samples were resuspended in 100 μl of 95:5 water:acetonitrile and 5 μl of this sample removed to a fresh vial and then diluted with 195 μl of 95:5 water:acetonitrile to provide an effective forty-fold dilution of the original extract. Both sample dilutions were then transferred to HPLC vials for CCM analysis by injection onto the HPLC-MS/MS system as described in Medina-Torres *et al*.^60^.

### Assessment of mitochondrial function

Sequential maximal electron flow through different complexes of the mitochondrial electron transport chain was assessed in uncoupled mitochondria using a SeaHorse XF24 analyser in n = 3 in the *db*/*m* and n = 5 mice/group in the *db*/*db* with and without treatment. Briefly, 10 µg/well of cortical mitochondria per mouse were loaded in triplicate into a 24-well cell culture plate and spun at 2,000 × *g* for 20 min at 4 °C. Mitochondrial oxygen consumption was measured in the presence of complex I substrates glutamate (10 mM) and malate (4 mM) and the uncoupler carbonyl cyanide-4-(trifluoromethoxy)phenylhydrazone (FCCP; 4 µM) for 10 min. Sequential injections of rotenone (2 µM, inhibits electron flow at complex I), succinate (10 mM, complex II substrate), antimycin A (4 µM (inhibits electron flow at complex III), and ascorbate:N,N,N’,N’-tetramethyl-p-phenylenediamine (10 mM:100 µM) were used to calculate electron flow at complex I, II, II and IV, respectively of the electron transport chain.

### Statistical analyses

Kolmogorov-Smirnov and Shapiro-Wilk Tests were applied to the data to assess normality. Parametric data were analyzed by one-way ANOVA with Tukey’s post hoc correction. For non-parametric data, Kruskal-Wallis one-way ANOVA with Dunn’s post hoc correction was performed. Where stated in figure legends, Student’s unpaired *t*-test or Mann-Whitney U tests were performed for comparisons between two groups. Data are expressed as mean ± SD, unless otherwise stated. *P* < 0.05 was considered statistically significant. Metabolomics data were analysed by multivariate analysis methods using SIMCA (MKS Umetrics AB, Sweden). Data were initially analysed using Principal Component Analysis (PCA) and subsequently by orthogonal projection to latent structures-discriminant analysis (OPLS-DA) where appropriate.

## Electronic supplementary material

Supplementary Information
