# Supplementary material for: Targeted mitochondrial therapy using MitoQ shows equivalent renoprotection to angiotensin converting enzyme inhibition but no combined synergy in diabetes
Source: Sci Rep. 2017 Nov 9;7:15190. doi: 10.1038/s41598-017-15589-x (PMC5680236; doi:10.1038/s41598-017-15589-x)
Supplement: Supplementary file 1 — Supplementary Information [file 41598_2017_15589_MOESM1_ESM.pdf]

Online Supplemental Material – SREP-17-29123A

Targeted mitochondrial therapy using MitoQ shows equivalent renoprotection to angiotensin converting enzyme inhibition but no combined synergy in diabetes

Micheal S Ward PhD#[1], Nicole B Flemming BSc(Hons)#[1,2], Linda A Gallo PhD[1,2], Amelia K Fotheringham BSc(Hons)[1,2], Domenica A McCarthy BSc(Hons)[1], Aowen Zhuang BBiomed(Hons)[1,3], Peter H Tang MD[6], Danielle J Borg PhD[1,2], Hannah Shaw [1], Benjamin Harvie[7], David R Briskey PhD[4], Llion A Roberts PhD[4], Manuel R Plan PhD[8], Michael P Murphy PhD[9], Mark P Hodson PhD[3,5,8] and Josephine M Forbes PhD*[1,3,10]

ID		Control		Diabetes		Diabetes + MitoQ		Diabetes + Ramipril		Diabetes + Combination	
		Average	SD	Average	SD	Average	SD	Average	SD	Average	SD
AAA_1	ASP	41.51675899	18.05861804	35.69960941	9.569456002	24.30426841	8.75221797	20.64294603	13.57033756	23.16702858	5.602214931
AAA_2	GLU	207.0709556	98.41805684	186.7725908	38.00384243	164.480947	48.54422131	168.3399415	62.93509829	201.2937824	46.33583574
AAA_3	CYS	72.11448545	33.61233026	37.31596974	14.36402273	41.92742347	14.29537677	50.31695116	25.09710155	39.84655274	19.36624975
AAA_4	ASN	2.280953611	0.850078238	2.234280819	1.503330246	2.086862281	0.489342757	2.25721686	0.966671958	2.792360279	1.853689088
AAA_5	SER	12.18004033	2.728690358	11.5822	3.011012147	10.96853488	1.996489837	10.61892764	3.366716981	12.35869001	4.86088752
AAA_6	GLN	38.26135334	18.19566684	26.82677372	8.787964002	21.28060592	3.419175149	23.64049727	12.13673876	29.46524408	8.197591924
AAA_7	HIS	4.372518387	1.501523213	4.310708788	0.983474683	3.641696229	0.663985375	3.713383689	1.255410787	4.442209104	2.011386883
AAA_8	GLY	154.2008027	45.87338807	89.32032264	10.69012009	94.00318667	16.71818868	79.26255456	43.20829471	90.50638649	22.45986204
AAA_9	THR	7.653772471	2.534215442	5.522101452	2.701889672	5.611224717	1.026325	6.327873108	2.433850332	7.08606652	3.773984844
AAA_10	ARG	2.479778543	0.941274455	2.894946336	0.982669279	2.827668084	0.669965586	2.990306664	0.925183571	3.782545346	1.657198438
AAA_11	ALA	49.19539793	18.45866685	54.64760832	25.70445301	46.10967047	13.28009089	52.14401625	19.21232792	63.36995534	39.41314259
AAA_12	TYR	4.327212	2.367761103	3.614965332	1.764790387	3.870906713	1.269387448	6.093590221	2.346630263	5.45933758	3.867743479
AAA_13	VAL	7.920195764	2.786018063	11.21022539	4.256361565	12.80356884	6.866504649	12.61074125	4.981724931	13.57809182	7.723444236
AAA_14	MET	1.989360774	0.950997171	1.215198875	0.721107007	1.48513185	0.454337914	1.628929967	0.728180962	1.873142721	1.231679563
AAA_15	TRP	1.206675509	0.559242493	1.20934052	0.255103086	1.367379514	0.412210628	1.739011826	0.579676146	1.777776776	1.002096258
AAA_16	PHE	3.546153048	1.30662199	3.373317205	0.731263335	3.492850059	0.515039758	3.870246765	1.17080875	4.638043865	2.53375725
AAA_17	ILE	2.914900921	1.280468709	4.043053628	1.519788725	4.995647619	2.854562051	4.605151604	1.899040395	5.082096516	2.857253798
AAA_18	LEU	7.401319952	2.759955153	9.451387537	3.639382913	10.91594709	4.585465225	11.14805921	3.893771411	11.55779973	5.91366241
AAA_19	LYS	4.799767159	2.231516463	3.999690827	2.452984963	3.493285141	0.893532581	4.366054624	1.898061067	5.542155539	4.546928042
AAA_20	PRO	3.072808847	1.432029652	2.346123737	1.351784675	2.270763763	0.724352468	3.286065216	1.487207555	4.095770996	4.25315879
CCM_1	ACO	0.226129212	0.047436666	0.355671581	0.061252501	0.277377637	0.139967337	0.28927411	0.103860725	0.367750292	0.167970963
CCM_2	ACoA	0.926577572	0.189180159	1.937450191	0.937032632	1.526589497	1.226336325	1.071104131	0.758674864	1.132439982	0.328713279
CCM_3	ADP	24.12345876	7.694734571	57.85473302	15.00600347	30.12709367	19.53714185	25.15759167	18.26336883	32.75578686	16.27788886
CCM_4	AMP	70.40333333	22.95208807	114.9783333	36.03375663	95.238	46.78991419	78.56	36.33420613	82.426	26.32232285
CCM_5	ATP	4.504582738	1.787246177	11.7865598	2.986847909	5.928485121	4.378014618	4.348678834	3.911976228	6.222060494	4.399367463
CCM_6	cAMP	0.028772508	0.011444836	0.042726958	0.009349336	0.029284703	0.009932457	0.02955793	0.011737566	0.03418348	0.004440752

CCM_7	CITISO	5.106494274	1.782530679	12.62222238	5.40724795	11.08281225	6.194463321	7.039784214	4.881663015	13.29162683	6.217115274
CCM_8	CMP	1.986543062	0.680007709	3.510500841	0.788661825	2.970237604	0.66168237	2.483931536	1.045300375	2.540309393	0.59145784
CCM_9	CPO4	5.0535	2.047422648	29.545	10.65166419	14.37948	15.34667892	10.80676667	12.10755274	12.8522	7.137898444
CCM_10	DHAP	0.507474783	0.181779767	0.727317429	0.168890074	0.466114074	0.080307271	0.740700104	0.290279265	0.660606255	0.310920977
CCM_12	F6P	1.256477197	0.322936329	4.352422656	1.393141722	2.293467116	1.493982322	2.436367526	1.501987472	3.302028649	2.066853431
CCM_13	FUM	8.079484845	3.31521535	5.9235418	3.206150003	8.617936397	5.293166131	9.718295483	3.550840708	15.07696889	6.50694893
CCM_14	G1P	0.084569773	0.015545044	0.170114097	0.040174925	0.104840092	0.017676669	0.173481762	0.059646616	0.154068863	0.042544226
CCM_15	G6P	0.619266365	0.159346575	1.665884627	0.454427437	0.840831965	0.51842392	1.016664464	0.529374449	0.872487522	0.356500797
CCM_16	GDP	3.197213445	0.076191845	4.30563763	0.720435958	3.912750045	1.327098301	3.409530398	1.251375266	3.608681401	0.722535518
CCM_17	Glycol	2.301070681	0.668305096	1.956189689	0.810622062	1.955708522	0.755755239	2.952186561	0.875410678	2.58178391	1.175431933
CCM_18	Glyox	4.139529119	0.841164728	3.08281678	0.428887582	3.816446124	1.543773539	2.882125876	1.26583302	3.746266935	0.355209769
CCM_19	GMP	10.41389218	2.155036822	14.11841566	2.911798549	12.39176459	4.097796887	11.53821583	3.817341625	12.08771929	2.153216237
CCM_20	KGA	0.297106819	0.143761483	1.247095733	0.479531169	1.215288152	0.686496576	1.335305001	0.603107887	1.617267568	0.827844557
CCM_21	LAC	1186.528269	750.1446225	1492.846651	500.1151014	1616.778218	530.5643809	2189.263428	964.8814153	3743.362818	4061.075596
CCM_22	MAL	93.94738282	39.78718067	102.3824262	31.02955601	121.8333558	62.40333068	103.9182069	40.640877	132.5031632	26.7244182
CCM_23	NAD	52.46807084	14.540193	92.25313481	19.84397401	69.24254618	32.65422708	63.68234806	26.55951214	73.07420317	17.64055295
CCM_24	NADH	2.613641205	0.545409883	5.112883571	1.75855522	4.931952428	1.635516926	3.547862843	1.767286881	4.04938541	1.390261263
CCM_25	NADP	4.295425999	0.805212891	8.153924353	2.225421385	5.188490616	2.42535582	5.723063203	2.38451678	5.294209146	2.600047577
CCM_26	PYR	1.160180183	0.723156501	2.118949001	0.729301432	2.025815254	1.183355012	2.29258461	0.909629312	2.384197874	0.514667736
CCM_27	R5P	1.07042069	0.2273758	1.111300025	0.198157562	1.329904861	0.887119735	1.108312891	0.483872385	1.175498965	0.076205257
CCM_28	RL5P	0.498282585	0.148488923	1.049618889	0.423014395	0.611847302	0.326705826	0.855131822	0.363895176	1.035118836	0.221518525
CCM_29	SUC	56.64633306	40.10892716	98.4355953	67.43141437	81.34693594	57.5045354	90.17156103	50.74292734	105.0537274	90.2597359
CCM_31	UDPG	10.28938895	4.874003481	14.31996627	1.240134349	13.41616232	2.661321377	12.33234145	4.117034689	14.19596615	0.872610993
CCM_32	UDPGA	6.503743781	2.726166309	8.255702702	3.646450398	4.058560512	3.231739758	6.309697221	3.157984353	4.873627287	3.222977626
CCM_33	UDPNAG	7.275489473	3.061683812	7.930016028	1.857409906	7.160871432	1.530784892	6.644040583	2.389452535	7.388166367	1.603264611
CCM_34	UMP	7.751384898	3.10455885	11.82663647	2.240547483	10.00798628	5.880890959	9.335179678	4.139278751	8.069425392	4.660658121

<u>Table S1:</u> Reference matrix of renal cortical metabolites and amino acids. Non-diabetic *db/m* mice (Control); diabetic *db/db* mice (Diabetes); diabetic *db/db* mice + 0.6 mg/kg/day MitoQ orally (DMitoQ); diabetic *db/db* mice + 3 mg/kg/day Ramipril orally (DRam); diabetic *db/db* mice + Combination therapy (DCoAd); n=6-8/group. Amino acids (AAA) were measured using HPLC. Standard abbrevations are used for amino acids and metabolites. All concentrations are in μmol/L.

	Control	Diabetes	DMitoQ	DRam
**Cortical Tissue**				
Ubiquinol-9 (nM/g)	215 ± 80	354 ± 166	364 ± 97	400 ± 194
Ubiquinone-9 (nM/g)	231 ± 82	434 ± 132	424 ± 111	394 ± 191
Total Q9 (nM/g)	446 ± 44	787 ± 133*	787 ± 94*	794 ± 237*
Ubiquinol-10 (nM/g)	33 ± 16	31 ± 15	30 ± 9	32 ± 14
Ubiquinone-10 (nM/g)	26 ±10	35 ± 17	30 ± 13	30 ± 17
Total Q10 (nM/g)	59 ± 8	66 ± 12	60 ± 8	62 ± 21
Total Q9:Q10 ratio	7.6 ± 0.8	12.0 ± 1.5*	13.2 ± 1.6*	13.4 ± 2.7*
**Isolated Mitochondria**				
Ubiquinol-9 (nM/g)	3313 ± 1787	3785 ± 2475	3521 ± 2279	4378 ± 1991
Ubiquinone-9 (nM/g)	5478 ± 963	7397 ± 1850	8169 ± 2746	7167 ± 2111
Total Q9 (nM/g)	8791 ± 1953	11182 ± 2789	11690 ± 4049	11545 ± 3142
Ubiquinol-10 (nM/g)	420 ± 192	348 ± 231	316 ± 201	398 ± 179
Ubiquinone-10 (nM/g)	1071 ± 166	875 ± 220	893 ± 229	799 ± 192
Total Q10 (nM/g)	1491 ± 335	1223 ± 355	1209 ± 364	1196 ± 282
Total Q9:Q10 ratio	5.9 ± 0.3	9.3 ± 0.7*	9.6 ± 1.1*	9.6 ± 0.7*

Table S2: Renal cortical tissue and isolated mitochondria analyzed by HPLC for oxidized (ubiquinone) and reduced (ubiquinol) coenzyme Q9 and coenzyme Q10 content. Non-diabetic *db/m* mice + Vehicle (Control); Diabetic *db/db* mice + vehicle (Diabetes); Diabetic *db/db* mice + 0.6 mg/kg/day MitoQ orally (DMitoQ); Diabetic *db/db* mice + 3 mg/kg/day Ramipril orally (DRam); n=4-10/group. FBG – fasting blood glucose; GHb – glycated haemoglobin. Data expressed as Mean±SD.
*P<0.05 vs C.
